# Hereditary Hyperferritinemia-Cataract Syndrome Misdiagnosed as Iron Overload: A Case Report

**DOI:** 10.7759/cureus.102469

**Published:** 2026-01-28

**Authors:** Serkan Güven, Menekse Öztürk

**Affiliations:** 1 Hematology, Çanakkale Mehmet Akif Ersoy State Hospital, Çanakkale, TUR; 2 Genetics, Çanakkale Mehmet Akif Ersoy State Hospital, Çanakkale, TUR

**Keywords:** cataract, hereditary, hyperferritinemia, iron overload, misdiagnosed

## Abstract

Hereditary hyperferritinemia-cataract syndrome (HHCS) is a rare autosomal dominant disorder caused by pathogenic variants in the iron-responsive element (IRE) of the 5′ untranslated region (5′UTR) of the FTL gene, resulting in dysregulated ferritin synthesis independent of body iron stores. Because elevated serum ferritin is commonly interpreted as a surrogate marker of iron overload, HHCS is frequently misdiagnosed as hereditary hemochromatosis or secondary iron overload, leading to unnecessary investigations and potentially harmful therapeutic phlebotomies.

We report the case of a 58-year-old male patient with longstanding unexplained hyperferritinemia, normal transferrin saturation, and a striking multigenerational family history of early-onset cataracts. Despite the absence of biochemical or radiological evidence of iron overload, the patient initially underwent therapeutic phlebotomy. Subsequent targeted sequencing of the FTL 5′UTR identified a heterozygous pathogenic c.-168G>A variant within the IRE, confirming the diagnosis of HHCS.

This case highlights a critical diagnostic pitfall in hematology practice and emphasizes the importance of interpreting serum ferritin in conjunction with transferrin saturation, exclusion of secondary causes, and careful assessment of family history. Early recognition of HHCS and appropriate use of targeted genetic testing can prevent inappropriate iron-depleting therapies and improve patient management.

## Introduction

Isolated hyperferritinemia does not always indicate iron overload. Elevated serum ferritin is a frequent laboratory finding in daily clinical practice and is commonly encountered during the evaluation of patients with suspected iron overload, chronic inflammation, liver disease, or malignancy. Because serum ferritin is widely used as a surrogate marker of body iron stores, its elevation often triggers further diagnostic investigations and, in some cases, therapeutic interventions aimed at iron depletion. However, ferritin is also an acute-phase reactant and may be increased in a variety of conditions unrelated to true iron excess, making its interpretation clinically challenging [1].

Hereditary hyperferritinemia-cataract syndrome (HHCS) is a rare autosomal dominant disorder characterized by persistently elevated serum ferritin levels in the absence of systemic iron overload and the development of early-onset bilateral cataracts. Although the exact prevalence of HHCS is unknown, it is considered exceptionally rare and is likely underdiagnosed. Since its first description in the mid-1990s, HHCS has been recognized as a paradigmatic disorder of iron metabolism regulation rather than iron accumulation. The condition is caused by pathogenic variants in the iron-responsive element (IRE) located within the 5′ untranslated region (5′UTR) of the FTL gene, which encodes the ferritin light chain [2,3].

Under physiological conditions, ferritin synthesis is tightly regulated at the post-transcriptional level by iron regulatory proteins (IRPs), which bind to the IRE under low-iron conditions and suppress ferritin translation. Mutations affecting the IRE disrupt this regulatory mechanism, leading to constitutive overproduction of ferritin irrespective of intracellular or systemic iron status. As a result, affected individuals exhibit isolated hyperferritinemia with normal transferrin saturation and no evidence of tissue iron deposition [4,5].

Despite its distinctive molecular basis, HHCS remains frequently overlooked in routine clinical practice. Elevated ferritin levels are commonly interpreted as evidence of hereditary hemochromatosis or secondary iron overload, particularly when early-onset cataracts or a suggestive family history are not actively sought. Consequently, patients may undergo extensive diagnostic work-ups and inappropriate therapeutic phlebotomy, which does not correct hyperferritinemia and may instead lead to iron deficiency anemia and unnecessary morbidity. This diagnostic pitfall is especially relevant in hematology clinics, where hyperferritinemia is often evaluated in the context of cytopenias, suspected malignancy, or systemic disease [5].

Reporting well-documented cases of HHCS, therefore, remains clinically important, as they reinforce the need for careful interpretation of ferritin levels and highlight the value of integrating biochemical patterns, clinical findings, and family history into diagnostic reasoning. The present case adds clinical value by illustrating a real-world example of delayed recognition and mismanagement in an adult patient with longstanding isolated hyperferritinemia, a striking multigenerational family clustering of early-onset cataracts, and molecular confirmation of a pathogenic FTL 5′UTR variant. This report underscores the importance of considering HHCS in the differential diagnosis of unexplained hyperferritinemia and aims to increase awareness of this rare but clinically significant condition.

## Case presentation

A 58-year-old male patient was referred to our hematology clinic for evaluation of persistent hyperferritinemia of unknown origin. Hyperferritinemia had been present for a long time according to the patient’s history, although the exact duration was unknown. A single session of therapeutic phlebotomy was performed due to suspected iron overload and an elevated hemoglobin level. His medical history was notable for bilateral cataracts, first diagnosed in the fifth decade of life, which showed gradual progression and ultimately required bilateral cataract surgery. Family history revealed multiple affected relatives, including his father, five sisters, his daughter, and a grandchild, all of whom had early-onset cataracts accompanied by elevated ferritin levels, suggesting an autosomal dominant inheritance pattern. A detailed multigenerational pedigree diagram could not be constructed due to the incomplete availability of clinical and genetic information from all affected family members. Nevertheless, the observed vertical transmission across successive generations strongly supports an autosomal dominant inheritance pattern.

Physical examination was unremarkable, with no clinical signs suggestive of iron overload. Laboratory evaluation revealed a hemoglobin level of 15.7 g/dL, a white blood cell count of 7.78 × 10⁹/L, a platelet count of 156 × 10⁹/L, and a mean corpuscular volume of 90.5 fL. Enzyme-linked immunosorbent assay-based viral serologies, including hepatitis B surface antigen, anti-hepatitis C virus antibody, and HIV testing, were all negative. Serum ferritin was markedly elevated at 790 µg/L (reference range 23-333 µg/L). In contrast, serum iron concentration, transferrin saturation, liver function tests, and C-reactive protein levels were within normal limits, excluding iron overload, hepatic disease, and inflammation-related hyperferritinemia (Table 1).

**Table 1:** Key laboratory findings and iron parameters at presentation HHCS: hereditary hyperferritinemia-cataract syndrome; AST: aspartate aminotransferase; ALT: alanine aminotransferase; CRP: C-reactive protein

Parameter	Patient Value	Reference Range	Interpretation in HHCS
Serum Ferritin (ng/mL)	790	23–336	Markedly elevated
Serum Iron (µg/dL)	95	50–178	Normal
Transferrin Saturation (%)	28	16–45	Normal
Total Iron Binding Capacity (µg/dL)	339	250–450	Normal
White Blood Count (10⁹/L)	7.79	4-10	Normal
Hemoglobin (g/dL)	15.7	12–16.8	Normal
Mean Corpuscular Volume (fL)	90.5	80–96	Normal
Platelet Count (10⁹/L)	156	100-400	Normal
AST (U/L)	22	8-40	Normal
ALT (U/L)	24	5-35	Normal
CRP (mg/L)	1	0-6	Normal

Abdominal ultrasonography demonstrated no hepatomegaly or evidence of hepatic iron deposition. The patient reported no history of alcohol consumption. Because of suspected hereditary hemochromatosis, a single session of therapeutic phlebotomy was performed prior to referral. This intervention did not result in any clinically meaningful reduction in serum ferritin levels, supporting the absence of true iron overload. Subsequent genetic testing revealed no pathogenic variants in the HFE C282Y or H63D loci.

**Figure 1:**
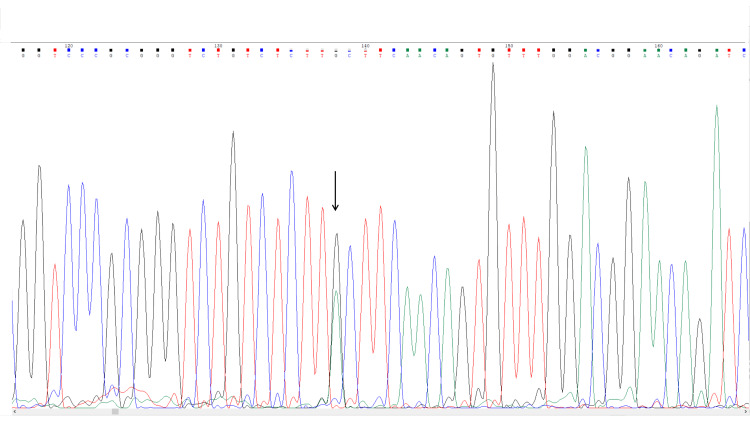
Sanger sequencing chromatogram of the FTL 5′ untranslated region showing the heterozygous c.-168G>A pathogenic variant. The arrow indicates the nucleotide substitution responsible for disruption of the iron-responsive element, confirming the diagnosis of hereditary hyperferritinemia–cataract syndrome.

Given the constellation of isolated hyperferritinemia, normal transferrin saturation, cataract history, and strong family history, HHCS was suspected. Targeted Sanger sequencing of the 5′UTR region of the FTL gene identified a heterozygous c.-168G>A (NM_000146.4) variant within the iron-responsive element. This variant has been previously reported in association with classic HHCS and is classified as pathogenic in the ClinVar database [6]. Based on clinical and molecular findings, a definitive diagnosis of HHCS was established. Following confirmation, first-degree and accessible extended family members were invited for genetic counseling and targeted FTL gene analysis (Figure 1).

## Discussion

HHCS represents a distinctive disorder at the intersection of iron metabolism, molecular genetics, and clinical hematology. Unlike conditions associated with true iron overload, HHCS is characterized by a marked dissociation between elevated serum ferritin levels and normal systemic iron indices. In this setting, serum ferritin reflects dysregulated ferritin synthesis rather than expansion of body iron stores, a distinction with major diagnostic and therapeutic implications [7].

One of the most important clinical features of HHCS is its frequent misdiagnosis as hereditary hemochromatosis or secondary iron overload. Because serum ferritin is commonly used as an initial screening marker for iron excess, elevated levels often trigger extensive diagnostic work-ups and iron-depleting interventions. However, transferrin saturation is a critical discriminator in this context. While typically elevated in hereditary hemochromatosis, transferrin saturation remains normal in HHCS, reflecting preserved systemic iron homeostasis [8].

Large case series and comprehensive reviews have demonstrated that HHCS is increasingly recognized worldwide but remains frequently misinterpreted as iron overload, leading to repeated and ineffective venesection therapy. Ferritin overproduction in HHCS is independent of iron availability; therefore, iron removal does not correct hyperferritinemia and may instead induce iron deficiency anemia and related clinical symptoms. These observations underscore that therapeutic phlebotomy in HHCS is not only ineffective but may also be potentially harmful [9].

Cataract formation is the most consistent and clinically recognizable manifestation of HHCS and may precede the diagnosis of hyperferritinemia by many years. The pathogenesis of cataract development is thought to involve intracellular accumulation of ferritin within the ocular lens, resulting in increased light scattering and lens opacity. Notably, the severity of cataracts and the age at onset do not correlate with serum ferritin concentrations, highlighting the limited prognostic value of ferritin levels in this disorder [10].

From a diagnostic perspective, HHCS should be considered in patients with persistent hyperferritinemia, normal transferrin saturation, and absence of inflammation, liver disease, or malignancy. A characteristic laboratory triad marked by hyperferritinemia, normal serum iron, and normal transferrin saturation combined with a personal or family history of early-onset cataracts should prompt consideration of HHCS and referral for targeted genetic testing. This diagnostic approach closely mirrors the clinical course of our patient [11].

Beyond individual patient management, recognition of HHCS has important implications for family members. Genetic counseling and cascade testing enable the identification of asymptomatic carriers, facilitate anticipatory guidance regarding cataract development, and prevent misinterpretation of ferritin elevations in affected relatives. Early diagnosis, therefore, contributes to personalized patient care and rational use of healthcare resources [12].

In conclusion, this case emphasizes that not all hyperferritinemia reflects iron overload. Awareness of HHCS and its characteristic biochemical and clinical profile is essential to avoid diagnostic errors and inappropriate treatment. Incorporating transferrin saturation, detailed family history, and targeted genetic testing into the evaluation of unexplained hyperferritinemia can substantially improve diagnostic accuracy and patient outcomes.

## Conclusions

HHCS is a rare but clinically important cause of isolated hyperferritinemia that is frequently misinterpreted as iron overload. This case underscores the critical importance of evaluating elevated ferritin levels in conjunction with transferrin saturation, inflammatory markers, and family history. Failure to recognize HHCS may lead to unnecessary investigations and inappropriate therapeutic phlebotomy, with potential harm to the patient. Increased awareness of HHCS among clinicians, particularly hematologists, and timely use of targeted genetic testing can prevent misdiagnosis, optimize patient management, and enable appropriate counseling and screening of affected family members.
